# Supplementary figures and images for: A data-driven model of macrophage polarization states reveals an IFN macrophage signature in active Crohn’s disease
Source: Front Immunol. 2025 Dec 4;16:1707719. doi: 10.3389/fimmu.2025.1707719 (PMC12711780; doi:10.3389/fimmu.2025.1707719)

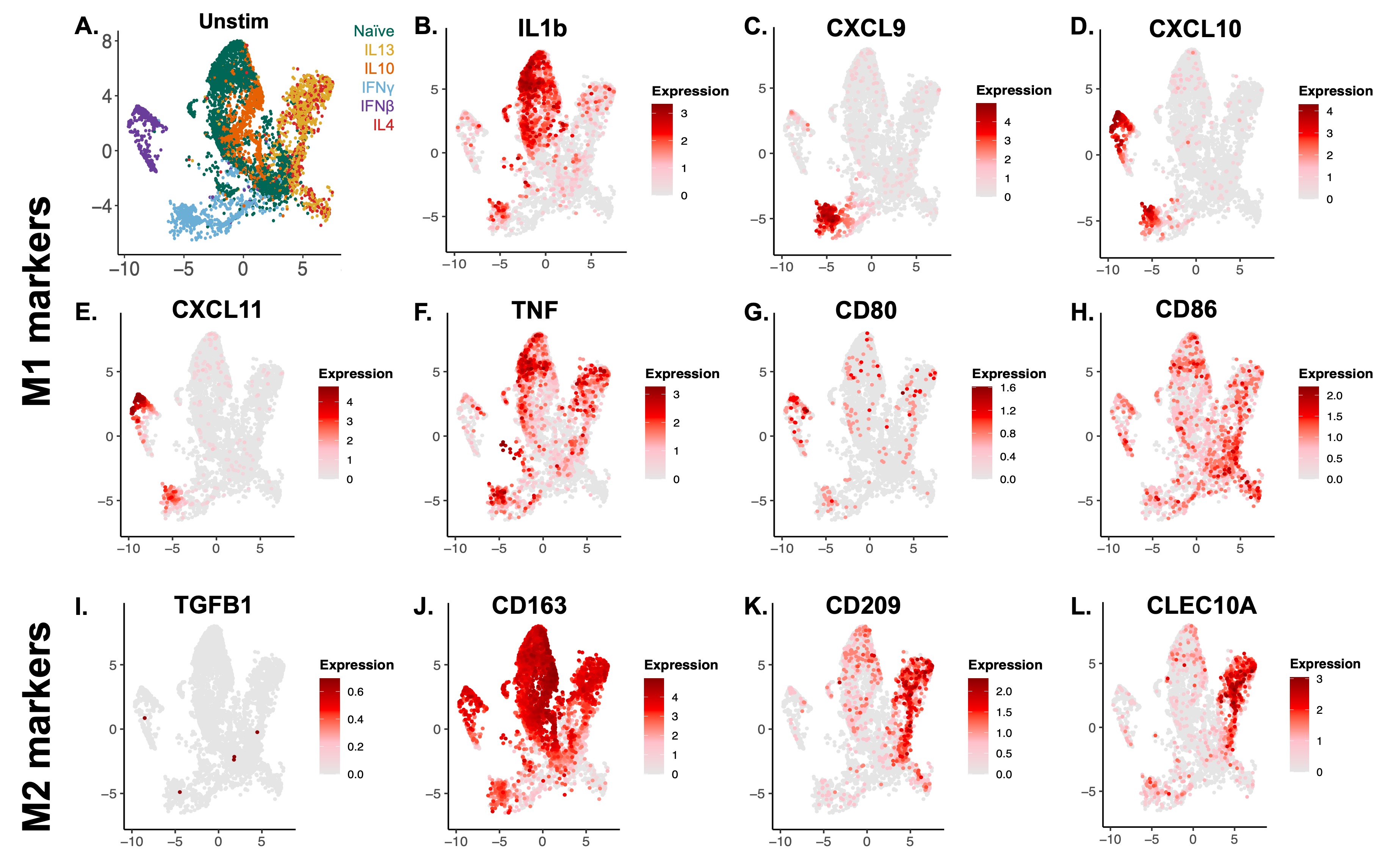

Supplement: Supplementary Figure 1 — M1 and M2 macrophage marker gene expression in polarized unstimulated macrophages. (A) UMAP of PBMC-derived day 6 macrophages polarized for 24 hours without acute stimulation. The samples are colored by polarization condition with naïve in green, IL-13 in yellow, IL-10 in orange, IFNγ in blue, IFNβ in purple and IL-4 in red. (This figure is the same as Figure 2A but shown here for reference). (B–H) Expression of M1 marker genes amongst the unstimulated in vitro polarized macrophages. (I–L) Expression of M2 marker genes amongst the unstimulated in vitro polarized macrophages. [file Image1.jpeg]

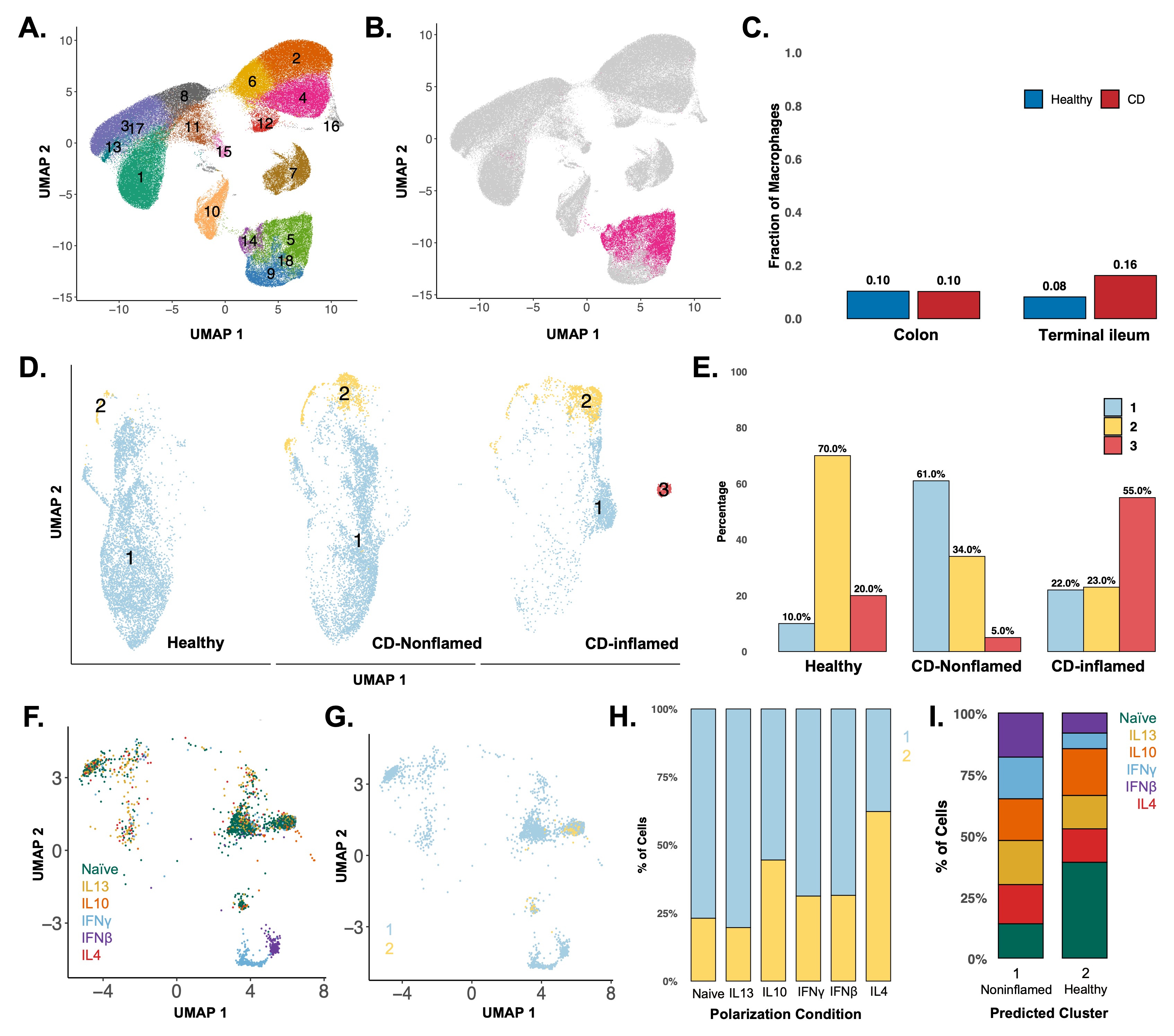

Supplement: Supplementary Figure 2 — Applying the data driven model of macrophage polarization states to colonic macrophages from Crohn’s patients. (A) UMAP of colonic immune cells from healthy donors and Crohn’s patients, from both noninflamed and inflamed regions. Immune cells were separated from stromal and epithelial cells based on annotations from the original study (Kong et al) and clustered in an unsupervised manner. The numbers delineate each individual cluster. (B) Immune cell types manually annotated based on the top 20 differentially expressed genes for each unsupervised cluster, with some clusters consolidated into broader cell type categories. The macrophage clusters are highlighted in pink. (C) Fraction of macrophages among immune cells from Crohns vs healthy donors in the colon vs terminal ileum. (D) Unsupervised clustering of the macrophage subset, separated by donor type. Cluster 1 is marked in blue, cluster 2 in yellow, and cluster 3 in red. (E) Distribution of macrophages assigned to each of the three clusters, separated by donor type. (F)In-vitro-polarized macrophages integrated with the colonic macrophage dataset using SCTransform anchors and mapped onto a shared UMAP, labeled by polarization condition. (G) Assignment of in-vitro-polarized macrophages to in vivo clusters using a weighted mutual nearest neighbor approach. (H) Proportion of cells from each polarization condition assigned to each in vivo macrophage cluster by mutual nearest-neighbor classification. (I) Assignment of polarization states to macrophages found in vivo which were grouped by unsupervised clustering (Supplementary Figure S2C) and are predominantly associated with noninflamed (cluster 1) and healthy (cluster 2). None were assigned to cluster 3. [file Image2.jpeg]
